# EEG decoding for effects of visual joint attention training on ASD patients with interpretable and lightweight convolutional neural network

**DOI:** 10.1007/s11571-023-09947-x

**Published:** 2023-03-07

**Authors:** Jianling Tan, Yichao Zhan, Yi Tang, Weixin Bao, Yin Tian

**Affiliations:** 1 Department of Biomedical Engineering, Chongqing University of Posts and Telecommunications, Chongqing, 400065 China; 2 College of Computer Science and Technology, Chongqing University of Posts and Telecommunications, Chongqing, 400065 China

**Keywords:** Autism spectrum disorder, Joint attention training, EEGNet, Feature visualization, Electroencephalogram

## Abstract

Visual joint attention, the ability to track gaze and recognize intent, plays a key role in the development of social and language skills in health humans, which is performed abnormally hard in autism spectrum disorder (ASD). The traditional convolutional neural network, EEGnet, is an effective model for decoding technology, but few studies have utilized this model to address attentional training in ASD patients. In this study, EEGNet was used to decode the P300 signal elicited by training and the saliency map method was used to visualize the cognitive properties of ASD patients during visual attention. The results showed that in the spatial distribution, the parietal lobe was the main region of classification contribution, especially for Pz electrode. In the temporal information, the time period from 300 to 500 ms produced the greatest contribution to the electroencephalogram (EEG) classification, especially around 300 ms. After training for ASD patients, the gradient contribution was significantly enhanced at 300 ms, which was effective only in social scenarios. Meanwhile, with the increase of joint attention training, the P300 latency of ASD patients gradually shifted forward in social scenarios, but this phenomenon was not obvious in non-social scenarios. Our results indicated that joint attention training could improve the cognitive ability and responsiveness of social characteristics in ASD patients.

## Introduction

Autism spectrum disorder (ASD) is a complex disorder of impaired neurodevelopment characterized by deficits in social communication and interaction that results in restricted behavior, interests, or activities (Amaral et al. 2018). The prevalence of ASD is reported to be about 1.5% in developed countries, and the large number of patients reflects its significant economic and social impact (Lyall et al. 2017). Attention deficit is one of the main symptoms of ASD, and the ability to pay attention can directly affect the efficiency of visual search (Baron‐Cohen 1989). Due to the complexity and heterogeneity of ASD, the current understanding of its pathogenesis is incomplete. Therefore, attention training for ASD patients and exploring its effects at the electroencephalogram (EEG) level are valuable and significant.

Joint attention is the ability to develop early and respond to socialized visual information, which is defined as the ability of two people to focus jointly on the same object and to recognize another person's intention from gaze directions or other social attention cues (Bakeman and Adamson 1984). Previous studies have shown that ASD patients showed deficits in joint attention when gazing at others (Falck-Ytter and Von Hofsten 2011). Furthermore, it was reported that ASD patients preferred to watch non-social stimuli compared with social stimuli (Pierce et al. 2016), and to be less likely to look at the non-face portion of social scenarios (Sumner et al. 2018). Also, some studies have reported no differences in social attention between ASD patients and their normal peers (Van Der Geest et al. 2002). In the EEG domain, P300 was a valid and positive event-related potential (ERP) for studying attention, which was evoked by visual stimuli and dependent on attention, usually peaking 300 ms after the stimulus onset (Picton 1992). Researchers commonly used the oddball experimental paradigm to evoke the P300 to explore the cognitive properties of visual attention (Akaiwa et al. 2022; Friedel et al. 2020; Marhöfer et al. 2014). Thus, P300 has been widely used in the study of visual attention (Kamp 2020; Nan et al. 2018).

EEG signals involve multiple dimensions of information in the time, frequency and space domains. For decoding EEG signals, traditional machine learning algorithms use manual feature extraction, which may ignore much more discriminative information. In recent years, deep learning has been rapidly developing in the fields of speech (Yao et al. 2021), image (Wang et al. 2020a) and bioinformatics (Wang et al. 2020a). Here, convolutional neural networks (CNN) were widely used as a good decoding tool for EEG-based cognitive tasks (Hazra et al. 2021; Shen et al. 2020). Jiang et al. used a combination of CNN and gated recurrent unit to effectively extract the spatio-temporal features of EEG signals from ASD patients and achieved better classification results (Jiang et al. 2022). However, the visualization and the cognitive interpretability of features in the model were still insufficient. EEGNet was a compact convolutional neural network with a reduced number of fitted parameters (Lawhern et al. 2018). It used deep convolution and separable convolution to construct EEG features and classify them. Importantly, the resulting features were neuro-physiologically interpretable. With limited training data, EEGNet still had a strong generalization capability and performance. However, few studies have applied EEGNet to the decoding of neural information in patients with ASD, especially in the visualization of decoding features for ASD attention training.

With the development of decoding technology, researchers were constantly updating experimental paradigms with new techniques to improve training outcomes for ASD patients, for example, combining virtual reality technology with brain–computer interface (BCI) for experimental training. In the research of Friedrich et al., they hypothesized that quantitative EEG-based neurofeedback training was feasible as a treatment option for ASD and used this hypothesis to propose an initiative to develop a gaming platform to achieve recovery of social skills through continuous training (Friedrich et al. 2014). Golan and Baron-Cohen suggested that the use of computer-based interventions in ASD patients can develop skills in a highly standardized, predictable, and controlled environment (Golan and Baron-Cohen 2006). Based on the above proposal, researchers created a visual attention training system for ASD patients that combined the advantages of ecological, realistic, and interactive virtual scenes with the attention-related properties of P300 and validated the reliability of the system experimentally (Amaral et al. 2017). For experiments in which a P300 signal could be evoked, researchers found that the central and parietal electrodes had stronger discrimination than the occipital and parietal-occipital electrodes (Brunner et al. 2010).

In this paper, we used the joint attention training data provided by Amaral (Amaral et al. 2017). The data sets contained two types of non-social characteristics and social characteristics for the study. Our study had two main goals: (1) To verify the effectiveness of EEGNet on EEG decoding before and after joint attention training in ASD patients. (2) To visualize the spatio-temporal features of EEG and the impact of joint attention training in ASD patients. Concerning these two goals, firstly, we classified EEG data of ASD patients by EEGNet. Then the feature classification contributions were visualized by saliency maps. Finally, the spatio-temporal features of EEG of ASD patients under joint attention training were visualized. It provided a new perspective to further understand the effect of joint attention training on the cognitive improvement in ASD patients, and provided new ideas for neurorehabilitation training methods for ASD patients.

## Materials and methods

For better reading experience, Table 1 showed the abbreviations and parameter symbols involved in this paper.

**Table 1:** Meanings and values of the abbreviations and parameter symbols

Name	Meaning	Value
ASD	Autism spectrum disorder	–
BCI	Brain computer interface	–
ELU	Exponential linear units	Activation Function
FL	Focal loss	Loss Function
CE	Cross entropy loss	Loss Function
ERP	Event related potential	–
LR	Logistic regression	–
$y$	Positive and negative samples truth class	$y \in \{ - 1,1\}$
${\text{p}}_{\text{t}}$	Predicted probability value	[0,1]
$\gamma$	Tunable attention parameter	2
$\omega$	Weight vector/derivative	Vary with back propagation
$b$	Bias	–
${M}_{(i,j)}$	Saliency map value	i-th row and j-th column

### Participants

Participants included 15 adolescents and adults with ASD ranging in age from 16 to 38 years with average age 22.2 years (Full-Scale Intelligence Quotient (FSIQ): Mean = 102.53; SD = 11.64). ASD participants inclusion criteria: (1) Positive diagnosis of ASD assigned according to the gold standard instruments. (2) Parental or caregiver interview (autism transmission). (3) Direct structured subject assessment (autism diagnostic observation schedule) (Lord et al. 1999). (4) The current diagnostic criteria for ASD according to the diagnostic and statistical manual of mental disorders, fifth edition (DSM-5) (Edition 2013).

Participants were excluded if they had intellectual disability, with a FSIQ below 80 (Wechsler 2008) and associated medical conditions such as epilepsy, neurocutaneous, or other genetic known syndromes, or other usual comorbidity in ASD samples (Amaral et al. 2018).

### Experiment setup

The virtual scene in the experiment contained a bedroom with objects (door, window, lamp, soccer, book, radio, printer, and laptop), furniture (bed, table, chair, shelf, and dresser), and one avatar. The BCI task was divided into three phases, the first two were offline, which were part of the BCI calibration process, and the last one was online.

In the first phase, participants were informed the target object directly to eliminate potential errors associated with the social attention deficit of ASD. In the second phase, participants were asked which objects the avatar had selected after it made an action to ensure that participants could read the cue correctly and were able to use this information correctly. In the third phase, participants were asked to respond to a cue from the avatar's head, namely, they were asked to imagine the avatar blinking at the stimulus target after focusing their attention during a trial. The first two phases had non-social characteristics, while the third phase had social characteristics.

**Fig. 1:**
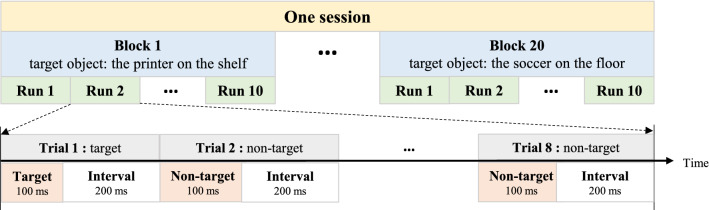
Diagram of the experimental composition of one session

In this experiment, all participants were required to do the same training session 7 times over a period of 4 months, the first four on a weekly basis and the last three on a monthly basis. Each session contained 20 blocks, each block consisted of 10 runs, and each run contained 8 trials in the virtual scene. During the experiment, the non-target stimulus or the target stimulus was flashed once at a fixed interval of 200 ms. Each flash lasted 100 ms. The experimental flow of one session was shown in Fig. 1. The EEG data acquired in session1 were considered as before joint attention training, and the EEG data acquired in session 7 were considered as after joint attention training.

Each block had one fixed target object (target object: the printer on the shelf, the laptop on the table, the soccer on the floor, the photo on the wall, etc.) with a total of 80 trials. The first 20 trials were calibration trials, which recorded EEG data including the P300 response generated when the target object flashed. Statistical classifiers were trained to identify P300. These classifiers were used in the online phase to identify whether the participant was responding to the flash of the object that the avatar interested. If the participant completed the action correctly, the BCI gave positive feedback (the object of interest turned green at the end of the trial). If not, that target object turned red.

### Data acquisition and preprocessing

Experiments were conducted using the P300-based virtual reality BCI paradigm. Immersive virtual scenes were presented to participants via the Oculus Rift Development Kit 2 headset, and participants were required to wear both the headset and a 16-electrode wireless EEG cap during the experiment. Here, EEG data were recorded from the same 8 electrodes positions (C3, Cz, C4, CPz, P3, Pz, P4, POz). The reference position and the ground position were placed at the right ear and the AFz, respectively, and the sampling rate was set to 250 Hz. To improve the signal-to-noise ratio of the signal, a 50 Hz trap filter was used during acquisition of the EEG data. Then, the EEG data were band-pass filtered from 2 to 30 Hz, followed by segmentation of the data from 200 ms before stimulus onset to 1200 ms after stimulus onset.

Since the interval between two trials in the experimental design was only 300 ms, the two trials data before and after the appearance of the target stimulus were censored separately in this study in order to avoid the influence of the P300 component on the target stimulus. The labels in the data were defined as “1” for the target stimulus and “0” for the standard stimulus. The EEG data were also detrended and extracted from 100 ms before the stimulus onset to 700 ms after the stimulus onset, and the shape of the processed data was 8 * 201.

### Model structure

**Fig. 2:**
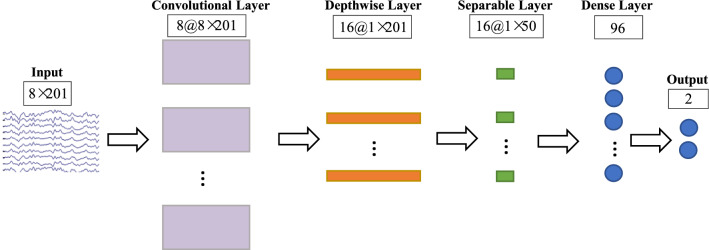
Architecture of EEGNet in the present study. White boxes displayed the information of the corresponding layers. The number before “@”: the number of filters per layer. The size after “@”: input data size for per layer. The rectangle indicated the convolution and the circle indicated the result after flattening. Different sizes represented different input sizes of these layers. Different colors represented different convolution kernel parameters were set

To ensure the accuracy of decoding the P300 signal contained in the EEG of ASD patients, this study used EEGNet, which was validated in a previous study, as a classifier (Lawhern et al. 2018). EEGNet was a lightweight neural network with a strong generalization capability for EEG data processing. Figure 2 showed the model architecture of EEGNet, and Table 2 described the parameters of each layer of the network model applied in the current study.

**Table 2:** Detailed parameters for EEGNet in the present study

Block	Layer	Number of filters	Kernel	Output dimension	Activation function
1	Input			8 × 201	
	Reshape			1 × 8 × 201	
	Convolution	8	1 × 64	8 × 1 × 201	
	BatchNorm			8 × 1 × 201	
	Depthwise Convolution	16	8 × 1	16 × 1 × 201	
	BatchNorm			16 × 1 × 201	
	Activation			16 × 1 × 201	ELU
	Average Pool		1 × 4	16 × 1 × 50	
	Dropout			16 × 1 × 50	
2	Separable Convolution	16	1 × 16	16 × 1 × 50	
	BatchNorm			16 × 1 × 50	
	Activation			16 × 1 × 50	ELU
	Average Pool		1 × 8	16 × 1 × 6	
	Dropout			16 × 1 × 6	
	Flatten			96	
	Dense			2	Softmax

In applying EEGNet, it was necessary to create a two-dimensional array of EEG data, where channels (8) and time (201) were represented by rows and columns of data, respectively, so that the shape of the input data was 8 * 201. EEGNet had two main blocks (refer to Table 2), 8 temporal filters and 16 spatial filters. The first block had convolutional filters whose kernel size was 1 * 64 to generate temporal feature mappings. Based on that, the spatial features were learned using depthwise convolution (Zhang et al. 2019) with a kernel size of 8 * 1. In this case, the number of spatial filters learned for each feature map was twice the number of temporal filters. After applying the temporal and spatial filters, the output features were normalized and exponential linear units (ELU) (Clevert et al. 2015) was used as the activation function. The distribution of the ELU output was zero-mean for faster training and unilateral saturation for better convergence. It was expressed as Eq. 1:

1 $$ f\left( x \right) = \left\{ {\begin{array}{*{20}c} x & {{\text{when}}\;x \ge 0} \\ {e^{x} - 1} & {{\text{when}}\;x < 0.} \\ \end{array} } \right. $$

To reduce the dimensionality of the features, an average pooling layer with a step size of 4 and a kernel size of 1 * 4 was used to dimensionality reduction of the features. Also, to prevent overfitting, the dropout (Srivastava et al. 2014) function was used to deactivate the neurons with a certain probability. After the above steps, the shape of the output features of the first block was 16 * 1 * 50.

In the second block, 16 filters with a separable convolution of 1 * 16 kernel size were used to learn the depth-time features of the EEG signal. Since the separable convolution had fewer parameters than the normal convolution, the model was less prone to overfitting. Again, the ELU activation function was used after batch normalization, and then the features were downscaled using an average pooling layer with a step size of 8 and a kernel size of 1 * 8, and the dropout function was used to alleviate the overfitting problem during training. Finally, a dense layer with a softmax activation function was used to classify the data. Softmax was used in the multi-classification process, it mapped the data to the interval [0,1] and constructed the output value as a probability distribution. The larger the mapping value of the softmax activation function, the greater the probability of the true category. The formula for calculating the softmax function was given below (Eq. 2):

2 $$ f\left( x \right) = \frac{{e^{{x_{i} }} }}{{\mathop \sum \nolimits_{i} e^{{x_{i} }} }}\quad {\text{for}}\;i = 1, \ldots ,n $$

In this paper, EEGNet was implemented using the Tensorflow framework, and Adam was chosen as the optimizer. The model learning rate was set to 1.25 × 10^−4^, β_1_ = 0.9, β_2_ = 0.999. The batch size for the mini-batch gradient descent was set to 128 examples, and the number of iterations was set to 300, while the neural network was trained using an early stop strategy. In addition, the network learned network parameters by back propagation. Due to the imbalanced number of positive and negative samples, we used focal loss as the loss function for training. The model was trained using the leave-one-subject-out method on NVIDIA GTX 1080, which meant that each subject's data were used for testing, the other remaining subjects' data were the corresponding training set. For a total of 15 subjects in this study, 15 training sessions and 15 testing sessions were required to obtain the corresponding classification accuracy on each subject's data. Finally, the average accuracy and the standard deviation of the classification accuracy obtained from 15 subjects’ test results.

### Loss function

Since the experiment was designed using the oddball paradigm, it resulted in a dataset with an imbalance of positive and negative samples [positive samples (target): negative samples (non-target) = 1:7] obtained in this experiment. Because of the large difference between positive and negative samples, this led to the difficulty of the neural network to learn features with a small number of positive samples, making the data with a small number of positive samples frequently misclassified and reducing the classification accuracy. And focal loss (FL) was proposed to solve the problem of sample imbalance (Lin et al 2017).

Focal loss was used in the classification task of the present study. The purpose of focal loss was to solve the problem of data imbalance between positive and negative samples, which was improved by balancing the cross-entropy (CE) loss. We introduced the focal loss starting from the CE loss (Eq. 3) for binary classification. The following mathematical formula was used to express the expression of focal loss in a rigorous way.

3 $$ {\text{CE}}\left( {p, y} \right) = \left\{ {\begin{array}{*{20}c} { - \log p} & {{\text{if}}\;y = 1} \\ { - \log \left( {1 - p} \right)} & {{\text{otherwise}}{.}} \\ \end{array} } \right. $$

where $y \in \left\{ { \pm 1} \right\}$ denoted positive and negative samples and $p \in \left[ { 0,1} \right]$ denoted the probability estimated by the model for the class with label *y* = 1. For notational convenience, we defined ${p}_{t}$ as Eq. 4:

4 $$ p_{t} = \left\{ {\begin{array}{*{20}c} p & {{\text{if}}\;y = 1} \\ {1 - p} & {{\text{otherwise}},} \\ \end{array} } \right. $$

5 $$ {\text{and}}\;{\text{rewrote}}\;{\text{CE}}\left( {p, y} \right) = {\text{CE}}\left( {p_{t} } \right) = - \log \left( {p_{t} } \right) $$

In the process of model training, the cross-entropy function treated the number of samples in each category as the same. However, the number of samples in each category was not always balanced in the model training process, and the cross-entropy weights each sample the same, which led to the category with more samples occupying most of the loss and their dominating the direction of model optimization. Eventually, it would lead to the classification effect of the hard-to-classify samples was not optimized.

To solve the above problem, a modulation factor ${\left(1-{\mathrm{p}}_{\mathrm{t}}\right)}^{\upgamma }$ was added in front of the CE loss, where γ was called tunable attention parameter, and γ ≥ 0. Therefore, the focal loss was defined as Eq. 6:

6 $$ {\text{FL}}\left( {p_{t} } \right) = - \left( {1 - p_{t} } \right)^{\gamma } \log \left( {p_{t} } \right) $$

Therefore, two properties of the focal loss were noted: (1) When the sample was misclassified and ${p}_{t}$ was close to 0, the modulation factor was close to 1 and the FL was unaffected. As ${\text{p}}_{\text{t}}$ neared 1, the factor neared 0 and the FL for well-classified examples was down-weighted. (2) When γ = 0, FL was equivalent to CE, and as γ was increased the effect of the modulation factor was likewise increased. It was found that γ = 2 worked best in previous studies (Lin et al 2017), so we chose γ = 2.

### Saliency map

Neural network had the ability to automatically extract features, and the visualization of neural network could facilitate the understanding of the EEG features learned by EEGNet. These features drove the model to make the appropriate decisions. In this study, the saliency map was used as a visualization technique for networks (Arrieta et al. 2020), which was widely used in various fields. The saliency map (Simonyan et al 2013) was a representation of the features learned by the model in terms of gradients (output relative to the input). After training the model and fixing its weight, the gradients relative to the input were back-propagated back to the first layer of the neural network.

In the convolutional neural network, given an input EEG data *X*_0_, a class c, and a classification EEGNet with the class function $h_{c} \left( X \right)$, we would like to rank the values of *X*_0_ based on its influence on the $h_{c} \left( {X_{0} } \right)$. We started with a straightforward example. Consider the linear model for the class c as Eq. 7:

7 $$ h_{c} \left( X \right) = \omega_{c}^{T} X + b_{c.} $$

where the EEG data X was a representation of a one-dimensional vector form, $\omega_{c}$ and $b_{c}$ denoted the weight vector and bias of the model, respectively. From Eq. 7, it was easy to see that the magnitude of ω defined the importance of the corresponding X for the class c.

In deep neural network, the ${\text{h}}_{\text{c}}\left({\text{X}}\right)$ was a highly non-linear function of X, so the reasoning in the previous paragraph did not have good applicability here. However, the Taylor function was an approximate function that fitted well for deep neural network, so given an EEG data *X*_0_, we can approximate ${\text{h}}_{\text{c}}\left({\text{X}}\right)$ with a linear function in the neighbourhood of *X*_0_ by computing the first-order Taylor expansion with the expression as Eq. 8:

8 $$ h_{c} \left( X \right) \approx \omega^{T} X + b. $$

In the above Eq. 8, ω was the derivative of the function ${\text{h}}_{\text{c}}$ at *X*_0_. The expression was as Eq. 9:

9 $$ \omega = \left. {\frac{{\partial h_{c} }}{\partial X}} \right|_{{X_{0.} }} $$

here the classification was based on the similarity of the input data for each category. The magnitude of the derivative ω indicated which *X*_0_ need to be changed the least to affect the class result the most.

In this paper, the derivative ω was found by back propagation. After that, the saliency map was obtained by rearranging the elements of the vector ω. The number of elements in ω was equal to the number of pixels in X_0_, so the map can be computed as $M_{(i,j) } = \omega_{(i,j)}$, where $(i, j)$ was the index of the element ω, corresponding to the pixel in the i-th row and j-th column. We used the saliency map method for within-subject feature visualization. First, the within-subject saliency map was calculated for data from the same cognitive task. Then, the resulting saliency maps were normalized. Next, the normalized saliency maps were averaged by superimposing them. Finally, the average saliency map of all subjects was obtained.

## Results

### EEGNet performance

In this paper, the intra-subject decoding of EEG data for each session in non-social and social scenarios was performed by EEGNet, respectively. Details of the non-social scenarios decoding accuracy were shown in Table 3. The average decoding accuracy before and after training was 86.3 ± 4.7% and 82.6 ± 4.6%, respectively. The social scenarios decoding accuracies were shown in Table 4, and the average decoding accuracy before and after training was 86.9 ± 3.9% and 85.9 ± 4.3%, respectively. In addition, the average accuracy of decoding in social tasks (86.2 ± 3.5%) was higher than in non-social tasks (84.5 ± 3.5) for ASD patients, and a paired t-test found this phenomenon to be significant (*t*(14) = 2.960, *p* = 0.01). The standard deviation here was calculated based on the classification accuracy of 15 samples and indicated the degree of dispersion of the sample data.

**Table 3:** Classification accuracy (%) of different sessions in non-social scenarios

Subjects	Session 1	Session 2	Session 3	Session 4	Session 5	Session 6	Session 7
1	83.5	79.3	81.1	80.6	71.4	76.9	81.5
2	88.8	85.2	91.2	86.4	88.2	86.3	89.3
3	88.2	89.3	90.0	86.9	75.5	74.1	72.4
4	87.4	87.6	76.9	84.2	84.4	83.2	83.1
5	94.6	91.1	89.4	84.1	88.7	89.3	88.3
6	86.9	85.8	82.7	85.7	90.5	84.9	86.3
7	83.9	84.0	86.9	83.3	86.9	81.3	82.2
8	81.7	92.4	85.2	92.9	91.1	80.9	82.3
9	86.9	83.4	80.4	82.3	84.6	75.7	78.6
10	93.4	92.3	90.4	77.7	97.6	87.5	87.5
11	88.7	92.9	85.5	92.9	89.9	85.1	80.6
12	79.6	73.4	88.7	81.7	83.9	80.1	84.6
13	80.7	81.1	83.8	79.7	77.9	91.1	85.1
14	79.8	77.2	78.2	82.0	78.1	75.7	75.7
15	90.6	91.0	84.6	84.0	86.3	85.7	81.2
Mean	**86.3**	85.7	85.0	84.3	85.0	82.5	**82.6**
SD	**4.7**	6.0	4.5	4.3	6.8	5.3	**4.6**

Mean indicates average decoding accuracy;

*SD* indicates standard deviation

**Table 4:** Classification accuracy (%) of different sessions in social scenarios

Subjects	Session 1	Session 2	Session 3	Session 4	Session 5	Session 6	Session 7
1	81.5	85.7	77.4	80.8	79.3	81.5	84.0
2	92.6	93.2	89.2	94.3	96.6	93.7	92.4
3	87.3	79.4	83.5	84.6	81.9	83.1	78.2
4	87.7	86.7	85.5	86.6	81.5	87.1	83.9
5	91.1	88.9	87.5	82.6	86.2	92.8	82.7
6	82.7	85.5	82.7	84.0	85.2	83.1	83.6
7	83.9	82.1	87.4	84.2	84.2	85.7	86.5
8	90.8	90.9	80.0	91.2	89.3	90.5	93.2
9	82.6	81.5	80.7	76.3	93.6	95.4	81.8
10	88.6	87.5	93.6	82.0	93.7	93.3	91.4
11	87.8	88.6	94.3	89.1	86.1	90.6	85.1
12	81.1	86.1	83.3	81.8	76.4	80.6	88.1
13	86.2	83.3	93.8	79.1	82.0	83.7	86.4
14	86.7	82.2	78.6	85.0	85.0	78.5	82.6
15	92.4	88.9	90.0	91.6	90.8	91.8	89.3
Mean	**86.9**	86.0	85.8	84.9	86.1	87.4	**85.9**
SD	**3.9**	3.8	5.6	4.9	5.7	5.5	**4.3**

Mean indicates average decoding accuracy

*SD* indicates standard deviation

Logistic regression (LR) was one of the most common model methods in the field of machine learning, which was often used as the baseline model for various tasks (Perlich et al. 2003). To provide a fair benchmark for EEGNet in this paper, we applied LR on a standard feature, the evoked potential P300, to classify between target and non-target. We performed an overlay average of the seven sessions for each subject and extracted the ERP mean of 350 to 400 ms as features. LR classification results showed that the average decoding accuracy before and after training in the non-social scenarios was 79.3 ± 1.3% and 77.6 ± 1.1%, respectively, and the average decoding accuracy before and after training in the social scenarios was 80.2 ± 0.9% and 78.5 ± 0.4%, respectively. Independent samples t-test showed that the average decoding accuracy of EEGNet (84.5 ± 3.5%) was significantly higher than that of LR (78.5 ± 1.0%) in non-social scenarios (*t*_(28)_ = 6.286, *p* < 0.001). In the social scenarios, the average decoding accuracy of EEGNet (86.2 ± 3.5%) was also significantly higher than that of LR (79.4 ± 0.4%) (*t*_(28)_ = 7.356, *p* < 0.001).

In terms of performance comparison, as shown in Table 5, the performance evaluation indicators for comparing the two models included the classification accuracy, sensitivity, specificity and the *p*-value of the t-test for accuracy. Here, sensitivity was the ratio of the number of test positives to the number of true positives, and specificity was the ratio of the number of test negatives to the number of true negatives. Table 5 showed that the sensitivity (87.7%) and specificity (84.0%) of the EEGNet model were stronger than the sensitivity (82.7%) and specificity (77.8%) of LR, respectively, in the non-social scenario. In the social scenario, the sensitivity (89.5%) and specificity (85.6%) of EEGNet model were also stronger than that of LR (85.2%) and specificity (78.6%), respectively. That was, in the same scenario, the sensitivity and specificity of EEGNet model were stronger than those of LR, so EEGNet had more prominent performance. It showed better classification accuracy and stability than the traditional LR method in the results, which also proved that EEGNet had a significant effect in the application to ASD.

**Table 5:** Classification results and performance comparison of LR and EEGNet

Model method	Scenarios	Accuracy (%)	Sensitivity (%)	Specificity (%)	***p* v**alue
LR	Non-social	78.5 ± 1.0	82.7	77.8	–
	Social	79.4 ± 0.4	85.2	78.6	–
EEGNet	Non-social	84.5 ± 3.5	87.7	84.0	*p* < 0.001
	Social	86.2 ± 3.5	89.5	85.6	*p* < 0.001

### Saliency maps from EEGNet

**Fig. 3:**
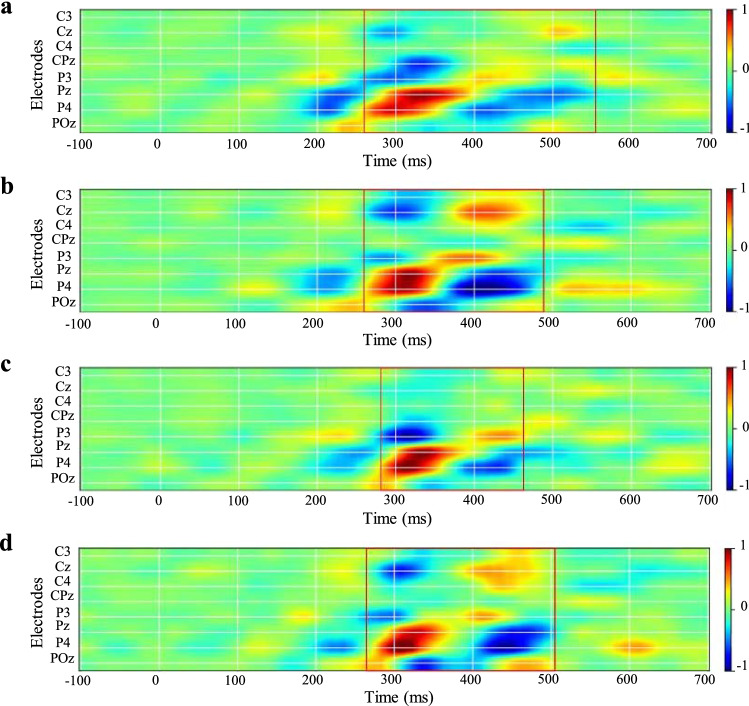
The within-subject average saliency maps. **a** Saliency map in non-social scenarios before joint attention training. **b** Saliency map in non-social scenarios after joint attention training. **c** Saliency map in social scenarios before joint attention training. **d** Saliency map in social scenarios after joint attention training. The maps showed the positive gradients in red and the negative gradients in blue

Figure 3 showed the within-subject average saliency maps for 15 subjects before and after training in both non-social and social scenarios, respectively. Figure 3a showed the saliency map in non-social scenarios before joint attention training. Figure 3b showed the saliency map in non-social scenarios after joint attention training. Figure 3c showed the saliency map in social scenarios before joint attention training. Figure 3d showed the saliency map in social scenarios after joint attention training.

From Fig. 3 we can see that the features driving EEGNet for classification were mainly concentrated in the parietal lobe at 300—500 ms after visual stimulus, such as P3, Pz, P4 and Cz electrodes. These features were consistent with the spatial distribution and temporal cycle of the P300 component. Over time, we could find that the largest contribution came from the 300—500 ms period, followed by the 200—300 ms period and around 600 ms. Each salient region could be interpreted as reflecting a neural process in the brain related to a cognitive task that was modulated by implicit attention at a specific time period.

Positive and negative gradients indicated the direction in which we would have to change this feature to increase the conditional probability of the attended class given this input example and their magnitude showed the size of this effect. The red rectangular box was a visual representation of the location and time period of the features with significant contributions.

### Visualization of spatio-temporal properties

**Fig. 4:**
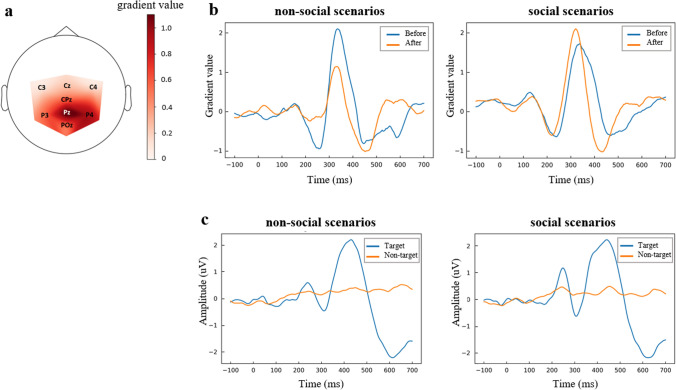
The results of the quantified spatio-temporal properties. **a** Spatial distribution of discriminative electrodes in decoding. The color bar represented average gradient value. The darker the color, the larger the gradient value, i.e., the most discriminatory of that electrode. **b** Temporal properties before and after attention training in two scenarios. **c** ERP of Pz electrode in two scenarios

The CNN model was particularly powerful when dealing with high-dimensional data because it had the ability to automatically extract task-relevant features from the data. Here, in addition to the analysis of the saliency map by visual inspection, quantitative analysis helped to further confirm the origin of the features. Therefore, this study quantified the spatial and temporal saliency maps by calculating the average of absolute values. The results of the quantified spatio-temporal properties were shown in Fig. 4

Here, we defined spatial properties as the most discriminative electrode during decoding throughout the experimental period. Because of the differences between ASD patients, we superimposed averaged the absolute values of the saliency maps for all subjects and the results were shown in Fig. 4a. The contribution of Pz in decoding was significantly greater than other electrodes. In terms of temporal properties, we explored them with the help of gradient values, and all electrodes were superimposed and averaged. The results were shown in Fig. 4b, and we can find that there was a gradient peak during 300—400 ms, which meant that the contribution of the signal during 300—400 ms was the greatest in the decoding process. Interestingly, the peak change in gradient before and after attentional training was different for ASD patients in different scenarios. Specifically, peak gradient before joint attention training was higher than that after training in non-social scenarios, and a paired t-test (before vs. after) showed that the difference was significant (*t*_(14)_ = 4.106, *p* = 0.001). However, in the social scenario, peak gradient before joint attention training was lower than that after training, and the peak difference was smaller. This suggested that ASD patients performed better in social scenarios with attention after multiple training sessions. But this training was not effective in non-social scenarios (*t*_(14)_ = −1.119, *p* = 0.282).

Pz as key position for decoding, we further checked ERP here. Similarly, we superimposed averaged the EEG data at Pz for all subjects, focusing on ERP differences brought by target in the same scenario. And the results were shown in Fig. 4c. As can be seen in Fig. 4c, there was a distinct positive wave around 400 ms after the onset of the target stimulus, i.e., P300, but the wave was not present during the non-target stimulus. In addition, there was also a significant positive wave around 200 ms after the onset of the target stimulus, i.e., P200. The P200 amplitude was stronger in social scenarios (*t*_(14)_ = 3.995, *p* = 0.001) and P300 was not significantly different (*t*_(14)_ = 0.196, *p* = 0.847) in ASD patients compared to non-social scenarios.

### Latency period of P300

The latency of P300 could be used to assess attentional cognitive ability, which was a potential marker of cognitive dysfunction. The waveform of P300 consisted of both amplitude and latency, with the amplitude of P300 representing the degree of information processing, which was related to the amount of attentional resources allocated to the task and the level of advanced cognition. Here, we calculated the amplitude of the most varied 100 ms for the EEGNet gradient to explore the variation in P300 latency. First, the saliency map was calculated for each subject at different sessions. Next, the saliency maps of 15 subjects at the same session were superimposed and averaged. Then, the intervals were quantified by a sliding window of 100 ms with a sliding step of 5 ms. Finally, the interval with the largest value was found to be the most varied 100 ms interval.

**Fig. 5:**
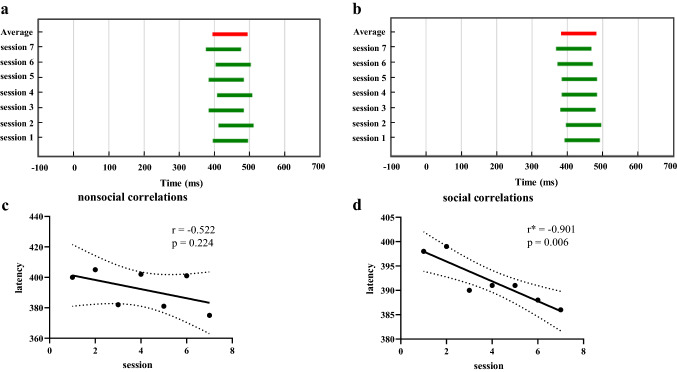
The most varied interval of the gradient. **a** The 100 ms interval in non-social scenarios. **b** The 100 ms interval in social scenarios. **c** Correlation between training times and P300 latency in non-social scenarios. **d** Correlation between training times and P300 latency in social scenarios. The dashed area was the 95% confidence interval

We selected the key electrode Pz for exploration. The results were shown in Fig. 5, where the most varied 100 ms intervals in non-social scenarios (Fig. 5a) and social scenarios (Fig. 5b) were located between 300 and 500 ms after stimulus onset. Focusing on the average interval showed that ASD patients had an earlier window in the social scenarios compared to the non-social scenarios, and a paired t-test of the average intervals in the two scenarios (non-social vs. social) for each subject indicated that the difference was significant (*t*_(14)_ = 2.905, *p* = 0.012). In particular, in social scenarios (Fig. 5b), as the number of training sessions increased, this varied interval was gradually shifted forward. Pearson method was used to test the correlation between training sessions and P300 latency. As shown in Fig. 5c, this trend of gradual forward movement was not obvious (*r* = −0.522, *p* = 0.224) in non-social scenarios. However, this forward shift phenomenon was obvious in social scenarios (Fig. 5d), which showed a significant negative correlation (*r* = −0.901, *p* = 0.006) between training sessions and P300 latency. It indicated that the P300 latency period became shorter and the response speed of ASD patients became faster, progressively. So social visual attention training had a positive effect on the cognitive status of ASD patients.

## Discussion

In the present study, we focused on the effects of joint attention training on ASD patients. First, we used EEGNet to decode the EEG signals of ASD patients and verified the effectiveness of EEGNet in decoding the P300 signal. Then, we visualized the cognitive properties of ASD patients during visual attention by saliency maps. Here, we found that the parietal lobe was the main area of classification contribution, especially for Pz electrode. And we also found that 300—500 ms produced the greatest contribution to signal classification, especially around 300 ms. Importantly, after training, ASD patients showed a significant boost in the gradient contribution at 300 ms, which was only valid in social scenarios. Moreover, with the increase in the number of joint attention training, the P300 latency of ASD patients was gradually shifted forward in social scenarios, but this phenomenon was not obvious in non-social scenarios. These findings indicated that joint attention training could improve the cognitive status of ASD patients.

In the EEGNet classification results in this paper, joint attention training of ASD patients had little effect on the overall classification, but EEGNet decoding performance was stable. Further compared with the results of LR, EEGNet had better performance. LR could be viewed as a simplified single-layer neural network. Although neural networks theoretically had the ability to approximate arbitrary functions, in practice it was difficult to train a single-layer network to have such ability. So EEGNet’s ability to learn features was better. Previous studies have reported the advantages of lightweight CNNs in BCI decoding, and the representative model EEGNet generalized well to both ERP and oscillation-based BCI (Lawhern et al. 2018). Therefore, it was beneficial to solve the visual attention decoding task by EEGNet for ASD patients, and the decoding performance of EEGNet in this paper was consistent with recent studies on EEG decoding (Borra et al. 2020). We also found that the average accuracy of decoding in social scenarios was higher than in non-social scenarios for ASD patients, suggesting that the feelings brought by social scenarios to ASD patients were special and more easily decoded by the model. Many studies have shown that attention training had a positive effect on attention in ASD patients, especially social attention (Cho and Ju 2018; Chukoskie et al. 2018; Spaniol et al. 2018). Wang et al. found that ASD patients looked less toward social stimuli (e.g., faces) compared to their non-ASD peers, limiting access to social learning (Wang et al. 2020b). Using gaze-contingent training, they found that the training effectively mitigated decreases of attention toward the face of onscreen social characters in ASD (Wang et al. 2020b).

EEGNet can directly process the raw high-dimensional data and automatically extract relevant features. If we visualized these features relevant to the cognitive task, there was an opportunity to further obtain information about the distribution of features when decoding the task. The saliency map can visualize the features extracted by the neural network at the individual trial level, at the within-subject level, and at the cross-subject level. In addition, saliency maps are not limited to variations in neural network architecture and are able to extract both spatial and temporal features. The results of the saliency map in this paper were shown in Fig. 3, where we found that the distribution of features was mainly concentrated in the parietal region and in the 300—500 ms time period after stimulus onset. This phenomenon was in agreement with the classical P300 component in terms of spatial distribution and temporal periodicity, revealing that the P300 is a meaningful ERP during the visual attention tasks. It was also similar to the results of Farahat (Farahat et al. 2019). They used the saliency map as a tool to visualize the spatial and temporal features driving the model output, and obtained the typical spatial distribution and latency expected for the P300 component during the task (Farahat et al. 2019).

The specific results for the spatio-temporal properties were shown in Fig. 4a and Fig. 4b. Among the series of electrodes in the parietal lobe, the most discriminating electrode was Pz. This was in line with the results of Krusienski et al. for the P300 speller (Krusienski et al. 2006, 2008). Their study targeted five electrodes (PO8, Cz, Pz, P3, and P4) and these positions were consistent with the distribution of P300 components (parietal lobe and central). In addition, the most discriminative time point was at about 300 ms after stimulus onset. After social scenarios training, the most discriminative time period was more concentrated to 300 ms and gradient value increased in ASD patients (by comparing the peak of the gradient before and after training). However, the opposite effect was observed after non-social scenarios training. This further suggested that ASD patients had different cognitive processes and different sensitivities to social and nonsocial scenarios. Many studies have also explored the differences in cognitive processes between these two scenarios for ASD and have demonstrated the value (Baker et al. 2020; Isaev et al. 2020; Keifer et al. 2021). For ASD patients who showed increased discrimination during 500—600 ms after joint attention training, this trend may have potential application in assessing and monitoring rehabilitation training modalities for certain neurological or psychiatric disorders.

For ASD patients, when a target object was present, the P200 amplitude was greater in social scenarios than that in non-social scenarios, as shown in Fig. 4c. It may be related to overt and covert attention. It has been reported in previous studies that for the overt attention task, the time period from 180 to 250 ms post-stimulus was the most informative, when P200 and N100 appeared. In contrast, for the covert attention task, the time period post-300 ms was the most informative, during which the P300 appeared (Treder and Blankertz 2010). When attention was overt, subjects focused on the target stimulus, and covert and overt attention modulate each other during the task (Lit et al. 2010). The results demonstrated that the P200 of ASD patients was more likely to be evoked in social scenarios and that P200 may be a biomarker of attentional modulation for ASD in visual search. Greater P200 amplitude might denoted that ASD patients were more sensitive to stimuli with social properties.

For P300 latency in ASD patients, we noted by Fig. 5 that latency was around 400 ms, which was correspond with ERP results in Fig. 4c. Previous studies have shown that P300 latency in normal subjects was 311.3 ± 37.0 ms (Hong et al. 2013). Hence, the P300 latency in ASD patients was longer than that in normal subjects. This longer latency period indicated that ASD patients had deficits in pre-information processing, resulting in a later response time point. In the 2 scenarios, the P300 latency became shorter in post-training (session 7) compared to pre-training (session 1), and Fig. 5 represented the most differential 100 ms interval shift forward. The trend of the forward shift in social scenarios was gradually evident with increasing number of training sessions, approaching the interval of normal subjects. This trend reflected that joint attention training had an improving effect on the cognitive state of ASD patients, demonstrating the effectiveness of the training from the EEG level.

It was also noteworthy that the achievement of EEGNet classification accuracy showed the promise of deep learning on disease datasets. These saliency maps could not only give us an indication of where and when to look for different neurologically relevant conditions, but also potentially provided an objective inspiration for conducting ERP studies. At the same time, it may be a useful direction for future research to combine the neural mechanisms of ASD patients to find more optimal rehabilitation training modalities.

## Conclusion

In this study, we analyzed the EEG data of ASD patients under the joint attention training experiment with the help of EEGNet and saliency map to examine effects of the training on ASD patients in social scenario versus non-social scenario. Our main findings of interest were as follows: (1) EEGNet was able to effectively decode the P300 signal of ASD and the EEG features learned by EEGNet had cognitive properties. (2) The spatio-temporal properties were manifested in the parietal region and 300—500 ms period, where Pz and 300 ms being the core node and core time point with the greatest decoding contribution. (3) ASD patients were more sensitive to stimuli with social characteristics. (4) ASD patients had delayed P300 latency, which can be improved with joint attentional training. More specifically, attentional training improved the attentional ability of ASD patients, and this improvement was more pronounced with society-characteristic stimuli. Therefore, visual joint attention training could improve the social cognitive level and responsiveness of ASD patients. Moreover, this study decoded the features of EEG in ASD patients with interpretability, which contributed to the further development of ASD research. It also provided a theoretical basis for the clinical treatment of ASD patients.

## Declarations

### Data availability

The data used in this study were provided by Amaral et al. (Amaral et al. 2018). The data set can be found here (https://doi.org/10.3389/fnins.2018.00477).
